# Commentary on: Muscle dysmorphia: Could it be classified as an addiction to body image?

**DOI:** 10.1556/JBA.3.2014.020

**Published:** 2014-12-18

**Authors:** JOHANNA E. NIEUWOUDT

**Affiliations:** School of Health and Human Sciences, Southern Cross UniversityLismoreAustralia

**Keywords:** muscle dysmorphia, classification, body dysmorphic disorder, behavioural addiction, body image, Diagnostic and Statistical Manual of Mental Disorders (DSM)

## Abstract

**Background:**

The article titled ‘Muscle dysmorphia: Could it be classified as an addiction to body image?’ used Griffiths (2005) addiction components model as the framework in which to define muscle dysmorphia (MD) as an addiction. The authors (Foster, Shorter & Griffiths, 2014) proposed that MD could be re-classified as an addiction to body image.

**Method and aim:**

In response to the original article, the author of this commentary reflected on the ‘Addiction to body image’ model and the components of addiction as described in the context of MD. This invited commentary aimed to provide opposing viewpoints in order to give a balanced overview on the topic.

**Results:**

It appears as if the components of addictions can be used as a framework in which to define MD. However, systematic empirical evidence had not been provided for the withdrawal symptoms associated with this behavioral addiction. An opposing viewpoint is provided in response to Foster et al.’s (2014) statement that MD is different from other body dysmorphic disorders in regards to cognitive dysfunction, and therefore cannot be explained in the same way.

**Conclusions:**

Based on the little systematic empirical evidence to date, it may be a bit premature to re-classify MD as an addiction to body image.

The article titled ‘Muscle dysmorphia: Could it be classified as an addiction to body image?’ by Foster, Shorter and Griffiths (2014) is a much-needed contribution to a discussion on muscle dysmorphia (MD) and its potential classification as a disorder. The authors proposed that MD could be re-classified as an addiction, with the addictive activity being the *“maintaining of body image”* (“An alternative classification”, para. 1). It is important to keep in mind that body image may have different meanings among various populations and cultures (Burlew & Shurts, 2013).

There is currently no agreement as to the specific meaning of addiction, including behavioral addiction (Karim & Chaudhri, 2012; Starcevic, 2013). Egorov and Szabo (2013, p. 199) described behavioral addictions as “compulsive psychological and physiological urges for one or more behaviour”. Only gambling addiction is currently included in the behavioral addiction category of the fifth edition of the *Diagnostic and Statistical Manual of Mental Disorders* (American Psychiatric Association, 2013). Grant, Potenza, Weinstein and Gorelick (2010) concluded that it is still premature to consider other behavioral addictions as independent disorders, especially since there are little or no validating data for proposed diagnostic criteria for behavioral addictions other than gambling addiction. It has been suggested that the symptoms associated with behavioral addictions are merely symptoms of other disorders (Karim & Chaudhri, 2012).

Apart from nosological issues, the practical implications of classifying MD as an addiction to body image need to be considered. There is currently no formal treatment for MD and no specific treatment studies have been conducted as yet. At this stage, treatment for MD can only be extrapolated from knowledge of related disorders (Grieve, Truba & Bowersox, 2009), such as body dysmorphic disorder (BDD), eating disorders and obsessive compulsive disorder. It is not known if MD will respond positively to the psychosocial and pharmacological treatments of behavioral addictions and substance use disorders.

Foster et al. (2014) used the components of addiction (Griffiths, 2005) as a framework in which to define MD. Each of the components of addiction was described in the context of MD. Salience referred to a total preoccupation with the particular activity (Griffiths, 2005), and had been identified by Foster et al. (2014) as activities that maintain body image such as physical exercise and specific eating habits. If the individual is unable to engage in the addictive behavior, reverse salience will occur, causing the behavior to become the most important thing in the individual’s life (Griffiths, 2005). Foster et al. (2014) stated, somewhat speculatively, that if the individual is unable to work out, it may result in physical symptoms such as sweating, shakes, and nausea, similar to those seen in other addictions. Furthermore, the authors stated that fainting and unconsciousness may occur due to low blood sugar levels, as a result of some of the dietary restrictions that may be present. This seems unlikely, as the highly structured dietary plans of weight lifters typically consist of copious amounts of healthful foods (Parent, 2013).

Mood modification referred to the ability of the maintenance behaviors to modify the mood of the individual (Griffiths, 2005). Foster et al. (2014) stated that weight lifting and eating (either restrictive or over-eating) can increase positive mood. This is achieved as a result of the release of endorphins into the bloodstream, thereby creating a chemical high (Foster et al., 2014). The authors further suggested that these chemical changes in the blood plasma may have the same effect as other psychoactive substances. Although the level of endorphins in the plasma are usually higher during intense exercise (Dishman & O’Connor, 2009), endorphins cannot cross the blood-brain barrier due to its chemical structure (Berczik et al., 2012; Dishman & O’Connor, 2009). Consequently the increase of endorphins in the bloodstream may not have a direct influence on the brain (Berczik et al., 2012; Dishman & O’Connor, 2009), thus may not have the same effect as other psychoactive substances.

Tolerance referred to the increases in the amount of the activity that must occur over time in order to achieve the same effects as in the past (Griffiths, 2005). Foster et al. (2014) suggested that a change in training strategies and diet may achieve the desired effects. Furthermore, withdrawal will occur if the individual is unable to engage in the activity, causing the individual to experience negative physical and/or psychological effects (Foster et al., 2014). Foster et al. postulated that withdrawal symptoms may include “intense moodiness and irritability, anxiety, depression, nausea, and stomach cramps”, and that at least one or more of these symptoms will be experienced when the individual is unable to engage in the activity (Withdrawal section, para. 1). Research indicated that extreme anxiety may occur if the individual missed a workout session (Olivardia, Pope & Hudson, 2000), however, it is not known whether the other withdrawal symptoms, as stated by the authors, may occur.

According to the proposed MD criteria, MD is associated with clinically significant distress in occupational, social and other areas of functioning (Pope, Gruber, Choi, Olivardia & Phillips, 1997), thus conflict may occur if the individual prioritizes training and/or eating over all other activities (Foster et al., 2014). If an individual give up maintenance behaviors of training and eating for a period of time, they may relapse and return to the behaviors again (Foster et al., 2014).

Body image dissatisfaction had been found to be a feature of BDD (Cororve & Gleaves, 2001; Hrabosky et al., 2009; Rosen & Ramirez, 1998; Sarwer & Crerand, 2004), eating disorders (Hrabosky et al., 2009; Rosen & Ramirez, 1998), and MD (Choi, Pope, Olivardia & Cash, 2002). It appears that BDD sufferers are highly invested in their physical appearance, as several domains of body image were found to be negatively correlated with BDD severity (Didie, Kuniega-Pietrzak & Phillips, 2010).

Foster et al. (2014) explained that the maintenance behaviors, that is physical exercise and specific eating habits, may hide or mislead individuals away from the negative thought processes that are driving their addiction to body image. Foster et al. stated that “there is a difference in the cognitive dysfunction with a misconstrued self-body image compared to other BDDs”, and it is this cognitive dysfunction that causes individuals to think that they are not muscular enough (“An alternative classification”, para. 11). This might not be true for all individuals, as Olivardia et al. (2000) found that up to 42% of the participants in their study had “excellent” insight, while over half of the participants had “fair” insight into their MD symptoms.

It was also stated that MD cannot be explained in the same way as BDD, due to the cognitive dysfunction that occurs in the context of the potentially positive physical effects of exercise on the body, for example improvements in the shape, muscle tone, and/or overall health (Foster et al., 2014). However, one can speculate that it may be similar to individuals with BDD seeking and receiving cosmetic treatment for their BDD concerns. The cosmetic procedures should have a positive physical effect on the body, but cosmetic procedures do not always lead to an improvement in BDD symptoms. A research study found that 83% of participants with BDD had no change or an increase in BDD symptoms after cosmetic procedures (Phillips & Diaz, 1997).

While it can be argued that the negative perception of body image has the potential to become an all-consuming and damaging obsession, it may be a bit premature to pathologize body image. All of the components of addictions need to be present for a behavior to be defined as addictive (Griffiths, 2002), and there is, as yet, little systematic empirical evidence to support this particular addiction. As stated previously, it may be that the symptoms associated with behavioral addictions are merely symptoms of other disorders (Karim & Chaudhri, 2012).

